# Transcriptomic and Proteomic Tools in the Study of Hg Toxicity: What Is Missing?

**DOI:** 10.3389/fgene.2020.00425

**Published:** 2020-05-05

**Authors:** Cláudia S. Oliveira, Ana L. A. Segatto, Pablo A. Nogara, Bruna C. Piccoli, Élgion L. S. Loreto, Michael Aschner, João B. T. Rocha

**Affiliations:** ^1^Programa Pós-Graduação Stricto Sensu em Biotecnologia Aplicada a Saúde da Criança e do Adolescente, Instituto de Pesquisa Pelé Pequeno Príncipe, Curitiba, Brazil; ^2^Faculdades Pequeno Príncipe, Curitiba, Brazil; ^3^Departamento de Bioquímica e Biologia Molecular, Centro de Ciências Naturais e Exatas, Universidade Federal de Santa Maria, Santa Maria, Brazil; ^4^Department of Molecular Pharmacology, Albert Einstein College of Medicine, New York, NY, United States

**Keywords:** methylmercury, proteome, transcriptome, neurotoxicity, thiol, selenol

## Abstract

Mercury is a hazardous substance that has unique neurodevelopmental toxic effects in humans. However, the precise sequence of molecular events that culminate in Hg-induced neuropathology is still unknown. Though the omics studies have been generating an enormous amount of new data about Hg toxicity, our ability to interpret such a large quantity of information is still limited. In this opinion article, we will reinforce the necessity of new high throughput and accurate analytical proteomic methodologies, especially, thiol and selenol-proteome. Overall, we posit that improvements in thiol- and selenol-proteomic analyses will be pivotal in identifying the primary cellular targets of Hg. However, a better understanding of the complex cascades and molecular pathways involved in its toxicity will require extensive complementary studies in more complex systems.

It is well established that the chemical forms of Hg (Hg^0^, Hg^2+^, MeHg^+^, EtHg^+^) differ in their toxicokinetics and toxicodynamics (Syversen and Kaur, 2012). Moreover, the interaction of electrophilic forms of Hg with –SH or –SeH groups of the biomolecules can cause: (1) accelerated rate of inactivation of specific –SH– or –SeH–containing molecules (Oliveira et al., 2017, 2018; Kuras et al., 2018; Branco and Carvalho, 2019); (2) a decrease in the availability of Se for selenoprotein synthesis (Barbosa et al., 2017; Bjørklund et al., 2017; Oliveira et al., 2017, 2018; Ralston and Raymond, 2018; Nogara et al., 2019a,b); and (3) increased rate of Hg entry into cells (for example, MeHg-cysteine and cysteine-Hg-cysteine as a mimic of methionine and cystine, respectively) (Bridges and Zalups, 2010, 2017). Nonetheless, to better characterize the toxic effects of Hg, it is essential to understand its primary toxic target(s), which, in turn, will trigger a cascade of events culminating in cellular demise and pathophysiological changes. In this context, the advent of the omics approaches can be instrumental in unveiling primary toxic target(s) of Hg, and to identify the pathways activated in response to the interaction of Hg with these targets.

Based on the cationic Hg and –SH/–SeH chemical properties, it is well accepted that the primary Hg targets are the –SH/–SeH-containing proteins; however, our knowledge about which of them are the primary toxic target(s) and the precise molecular pathways involved in the toxicity of Hg remain elusive. In our opinion, both –SH and –SeH transcriptomic and proteomic analyses have to be taken into consideration in search of Hg’s primary toxic target(s). To our knowledge, the –SH–transcriptomic approaches cannot identify yet which of the cysteine codons (UGC and UGU; Rocha et al., 2017) will be free -SH in the nascent and functional proteins. However, the presence of a codon for cysteine in the mRNA point to a potential site for cationic Hg species binding. Indeed, any transitory free –SH group in a nascent protein (cysteinyl residues that will form a disulfide bond in functional proteins) are potential targets for cationic Hg species. On the other hand, the –SeH-transcriptomic analyses can provide a more precise view of potential targets for Hg-induced selenoprotein disruption, once practically all the selenocysteine residues will be in the –SeH form (Hatfield et al., 2014), i.e., free to react with cationic Hg species.

Despite the instrumental role that omics approaches may have in deciphering the targets and pathways involved in the toxicity of Hg, only a limited number of studies have evaluated the effect of Hg on the –SH– or –SeH-transcriptome and/or proteome and fewer made use of *in silico* analysis or artificial intelligence (AI) approaches. Until now, the transcriptomic data have provided valuable information on altered gene expression and/or regulation, but have failed to delineate whether these alterations are the cause or effect of Hg toxicity. Moreover, detailed studies on the time course of Hg-induced changes in gene expression, which are essential to figure out the first toxic target(s), have been rarely performed (Robinson et al., 2010; Theunissen et al., 2011). Therefore, the availability of the raw data in public databases is not mandatory for transcriptomic studies and several researchers have not deposited their data publicly, making this another limiting factor in the study of Hg toxicity. In addition, the detection of differential expression of specific sets of RNAs after exposure to very low to low levels of Hg (10^–21^ to 10^–13^ M) is highly needed to identify possible “expression signatures” that might be used as early markers of Hg exposure, much earlier than physiological or biochemical changes can be detected. Here, particularly for the very low levels of exposure, we will face additional analytical constraints, for instance, the necessity of new ultrasensitive methodologies capable of detecting trace quantities of R-Hg-proteins adducts. These limitations conceivably will require complementary omics- and non-omics approaches to be achieved.

mRNA and protein expression levels cannot be expected to correlate perfectly due to post-transcriptional regulation. Consequently, proteomic analyses can provide a more realistic view of the protein content in a determined developmental stage or pathophysiological condition (Fu et al., 2009), for instance, in the cases of Hg intoxication. In this context, the development of new proteomic techniques able to detect the primary toxic target(s) of cationic Hg species is pivotal.

The human selenoproteome and cysteine proteome were already determined (Jones and Go, 2011; Labunskyy et al., 2014; Go et al., 2015). Nonetheless, the development of reliable high throughput thiol-omics and selenol-omics analytical methodologies are highly needed to determine which of the proteins containing reactive and biologically relevant –SH and –SeH will interact with cationic Hg species. This will be instrumental in distinguishing proteins that are effectively involved in the genesis of Hg pathology from those that bind Hg but did not change the cell, tissue, and/or organism biological functions.

In the proteomic context, analytical methods have been developed to identify the redox-sensing thiols. The standard redox-sensitizing thiol workflow is based on (1) the treatment of the proteins with electrophilic compounds and, then, with thiol markers (labeling step), (2) a 2D gel electrophoresis is carried out, and (3) the selected proteins are separated from the gel and digested (proteolysis) to further LC-MS/MS identification. Cationic Hg species bound to proteins can be quantified by gel electrophoresis through the fluorescent labeling assay (for example, the BPM/biotin-PEAC5-maleimide labeling assay), where a decrease in the fluorescence reflects a decrease in the number of free –SH groups, i.e., an increase in the number of Hg-protein adducts (protein-S–Hg-R) (Toyama et al., 2013). In addition, with LC-MS/MS analysis, it is possible to identify cysteine residues to which Hg species are bound. However, the steps vary, depending on the methodology selected (Leichert et al., 2008; Hill et al., 2009; García-Santamarina et al., 2011; Jones and Go, 2011; Karlsen et al., 2014; Zaccarin et al., 2014). Nevertheless, concerning the –SeH proteomics, there are still no defined techniques. In the case of –SeH groups, the methodologies will have to take into account the very low level of occurrence and the extreme reactivity of the –SeH/–Se^–^ groups. The instability of the –C–Se– bond (Orian et al., 2015) in the Sec–Hg–R adducts is also of great analytical concern.

A persistent technical problem that compromises the identification of specific –SH groups targeted by Hg species is the necessity of denaturing the protein with detergents and reducing agents before the proteolysis, for instance, DTT or tris(2-carboxyethyl)phosphine (TCEP). This step is performed to unfold the protein and improve the fragmentation; however, the addition of high concentrations of –SH reducing agents can remove the cationic Hg species from proteins via an exchange reaction (protein-S-Hg-R + R-SH → protein-SH + R-S-Hg-R; Nogara et al., 2019b).

Of particular importance, the use of omics in toxicology can be limited by methodological uncertainties in interpreting and assessing data (Buesen et al., 2017). The use of transcriptomic and/or proteomic analyses is recent, and only a few studies have attempted to outline questions relevant to apical end-points of Hg toxicity. The majority of the studies that link differences in gene expression or protein activity/level with Hg toxicity have not used omics technologies. Indeed, omics technologies have been only employed in an exploratory way. The systematic application of omics analysis to simple relevant *in vitro* studies, followed by invertebrate models, will be crucial in generating more comprehensive and workable data capable of driving the elaboration of appropriate omics studies with vertebrates. A similar type of study to be followed as an example was carried out with manganese (Fernandes et al., 2019).

It will be crucial to explore different doses/concentrations of Hg, e.g., from very low (10^–21^ M) to high (10^–6^ M). As commented above, the analytical determination (R-Hg-protein adducts and/or total Hg) at very low Hg levels will be a challenge to be reached by the researchers; however, the cellular response can ideally be followed by cutting-edge cellular and molecular methodologies. Exposing cells to very low concentrations/doses of Hg for a short time will afford the opportunity to detect a possible compensatory hormetic mechanism, which can be interpreted as molecular responses resulting in cellular protection. With increased doses (concentrations) or exposure time, the compensatory mechanism can be exhausted, and the ensuing damage will manifest (Calabrese and Baldwin, 2001). These efforts will be pivotal in the understanding of the first signs of Hg toxicity and, consequently, will be helpful in the identification of the first target of cationic Hg species. A virtual protocol of exposure is depicted in Figure 1, using as an example methylmercury (MeHg^+^) the Hg organic chemical form ubiquitously present in fish.

**FIGURE 1:**
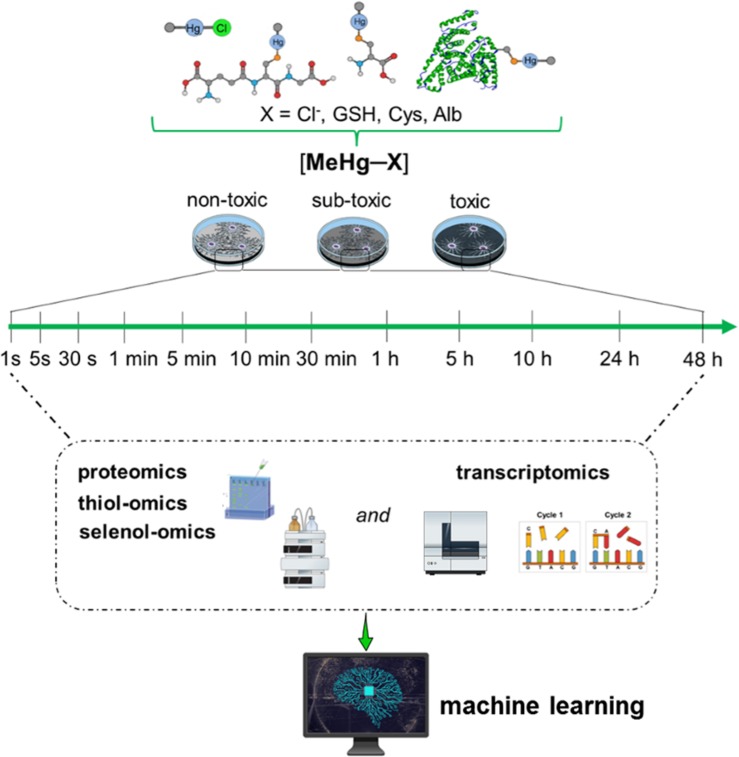
Hypothetical toxicogenomic studies using simple *in vitro* models. Here relevant human cell types have to be exposed from very low (10^–21^ M) to high (10^–6^ M) concentrations of MeHg^+^. For instance, non-differentiated or differentiated neurons or glial cells have to be incubated with MeHg^+^ and physiologically relevant MeHg-S conjugates (cysteine, GSH, albumin or hemoglobin, as an example see Tan et al., 2019) for different periods. MeHg^+^ toxicity will have to be determined by proteomics, specifically by the –SH– and –SeH-omics, epigenomics, metabolomics, and transcriptomics. In short, even for a single type of cell, the number of systematic analyses to be performed is arduous. They will allow the construction of a causal relationship between gene regulation/expression with protein and metabolites levels. Using these data (nucleotide and amino acid sequences) and AI algorithms, particularly machine learning, could be possible to identify the tertiary protein structure (e.g., thiol- and selenoproteins) and predict the motifs that will be more likely disrupted by MeHg^+^ facilitating the understanding of potential proteins involved in MeHg^+^ toxicity and consequently the first toxic target(s).

To better understand the enormous amount of data that will be generated from the time course and dose/concentration variation studies, the *in silico* analyses will be necessary. These types of analyses have been sparsely explored in Hg studies. The analyses have to include interaction modeling and pattern searching using AI algorithms, particularly machine learning and deep learning. In the future, machine learning should be used to identify/predict the tertiary protein structure from transcriptome (nucleotide sequence) and proteome (amino acid sequence) studies. Considering the scarcity of detailed tertiary/quaternary protein structures availability, *in silico* prediction models, will be crucial to identifying the preferential Hg binding sites, i.e., to construct models that can reveal the electrostatic environment of solvent exposed –SH and –SeH moieties. The identification of accessible and preferential Hg binding sites in tertiary predicted structures will allow identify electronic and chemical motifs that will be more likely disrupted by Hg, and which amino acids are more likely around these regions. The knowledge of such sequences will facilitate the identification of potential proteins involved in Hg toxicity. Upon identifying potential Hg target proteins using specific proteomic techniques (e.g., selenoproteins and thiol-containing proteins that have CC, CXC, and CXXC motifs), the use of knockout models in a systematic way will be useful for directly analyzing the role of specific thiol- and selenoproteins in the toxicity of Hg.

Thus, it is possible to conclude that to understand better the Hg cascade of events that will generate symptoms of toxicity, for example, irreversible symptoms, as observed in the Minamata disease, the researchers from different areas (Toxicology, Molecular Biology, Genetics, Biochemistry, and Bioinformatics) will have to join efforts. Punctual studies have been instrumental in understanding specific end-points of toxicity caused by Hg; however, only multidisciplinary studies will be able to figure out the first toxic target(s) of cationic Hg species. In addition, it will also be necessary to advance the knowledge of pathways and activation cascades in a physiological situation to understand the deregulation caused by xenobiotics, especially Hg, in more complex systems.

## Author Contributions

CO, AS, BP, and JR were responsible by the manuscript main idea. PN was responsible by the figure. ÉL, MA, and JR were responsible for the English grammar corrections. All the authors actively participated in manuscript writing.

## Conflict of Interest

The authors declare that the research was conducted in the absence of any commercial or financial relationships that could be construed as a potential conflict of interest.
